# Heavy Metals Content and Health Risk Assessment in Airborne Particulate from the Calabria Region, Southern Italy

**DOI:** 10.3390/ijerph21040426

**Published:** 2024-03-31

**Authors:** Francesco Caridi, Giuseppe Paladini, Maurizio Messina, Domenico Majolino, Valentina Venuti

**Affiliations:** 1 Dipartimento di Scienze Matematiche e Informatiche, Scienze Fisiche e Scienze della Terra, Università degli Studi di Messina, V.le F. Stagno D’Alcontres, 98166 Messina, Italy; dmajolino@unime.it (D.M.); vvenuti@unime.it (V.V.); 2 Dipartimento di Fisica e Astronomia “Ettore Majorana”, Università degli Studi di Catania, 95123 Catania, Italy; giuseppe.paladini@dfa.unict.it; 3 Dipartimento di Reggio Calabria, Agenzia Regionale per la Protezione dell’Ambiente della Calabria, 89135 Reggio Calabria, Italy; m.messina@arpacal.it

**Keywords:** airborne particulate, heavy metals, inductively coupled plasma mass spectrometry, hazard index, total cancer risk index

## Abstract

This study is focused on the determination of the heavy metals content in airborne particulate matter (PM) with a diameter lower than 10 µm (PM10) deposited on quartz microfiber filters and collected in four representative selected sites of the Calabria region, southern Italy. In particular, data on the content of Cd, Ni, and Pb in PM10 (i.e., those metals whose limit values, in terms of concentration, are reported in the Italian Legislation) were obtained through inductively coupled plasma mass spectrometry (ICP-MS) measurements after acid extraction with microwaves and filtration. Results showed that the average concentration of investigated metals decreases as Ni > Pb > Cd for all analyzed samples, and concentration values are lower than the limit values reported in the Italian legislation in all cases. Moreover, in order to assess the health risk related to their presence in the environment, the potential non-carcinogenic hazard for the investigated heavy metals was evaluated by calculating the hazard index (HI) for children and adults. Results indicated that the calculated HI values were lower than the safety limit in all cases, thus indicating a negligible non-carcinogenic health risk. In addition, the potential carcinogenic hazard for the investigated metals was estimated through the total cancer risk index (Risk_total_). Obtained results were also lower than the limit value for children and adults in this case, and, therefore, the carcinogenic health risk caused by heavy metals in the analyzed PM10 samples could be considered to be unremarkable.

## 1. Introduction

Airborne particulate matter (PM) consists of a broad class of chemically and physically different elements, varying in sizes, chemical compositions, formations, sources, and concentrations [1,2]. Exposure to PM has a negative impact on human health, and it contributes significantly to increases in premature deaths due to cardiovascular and respiratory diseases [3,4]. PM contains sulfates, nitrates, ammonium ions, hydrogen ions, other inorganic ions (e.g., Na^+^, K^+^, Ca^2+^, Mg^2+^, and Cl^−^), particle bound water, heavy metals, elemental carbon, and organic compounds [5,6]. The major urban causes of these PM-associated compounds are related to anthropogenic activities, such as mining, construction, industrial emissions, road traffic (motor vehicles, railways), various combustion processes, power plants, and domestic heating [7,8]. In particular, airborne particles with diameters lower than 10 µm (PM10) can affect climate and reduce visibility, as they participate in many significant atmospheric processes [9,10]. They are often harmful for health because, being able to overcome the protective barriers present in the first portion of the respiratory system, they can reach deeper areas [11]. The effects of PM10 are proportional to its concentrations, and there are no threshold values below which there is no danger to health. This is mainly due to the presence of carcinogenic compounds embedded in the particulate matter itself, such as heavy metals [12] (i.e., naturally occurring elements with relatively high density, atomic number, and atomic weight [13,14,15]). Their multiple uses in industry, housing, agriculture, medicine, and technology raise concerns about their possible impact on human health and the environment [16,17,18]. Heavy metals such as Cd, Ni, and Pb, also present at very low concentrations and have adverse effects on the human body, causing acute and chronic toxicity [19]. Therefore, for the protection of the environment and to ensure sufficiently clean air levels, heavy metals must be kept at safe levels [20]. In the last decades, a number of studies have been carried out in order to assess the levels of heavy metals in PM10 and their potential risks [21,22,23,24,25,26,27,28].

In view of the above, this study aims to determine the concentration of those metals whose limit values, in PM10, are reported in the Italian Legislation (i.e., Ni, Cd, and Pb) for four selected sampling sites spread across the entire Calabria region, southern Italy. It is worth noting that in this paper, for the first time, the assessment of the health risk associated with the presence of PM10 in the environment for children and adults residing in this region was carried out. This represents the absolute novelty of the present work, and obtained results could also be used for monitoring the elemental composition of atmospheric particulate matter, which can contribute to better air quality management.

## 2. Materials and Methods

### 2.1. Sampling

**Figure 1:**
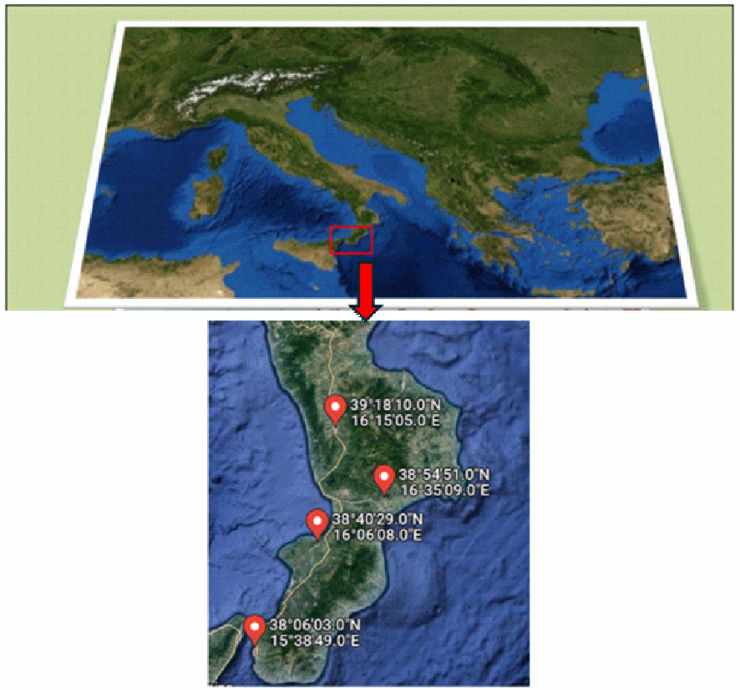
Map of the sampling points.

The selected sampling points are reported in Table 1 together with their identification code (IDs) and GPS coordinates and shown in Figure 1.

**Figure 2:**
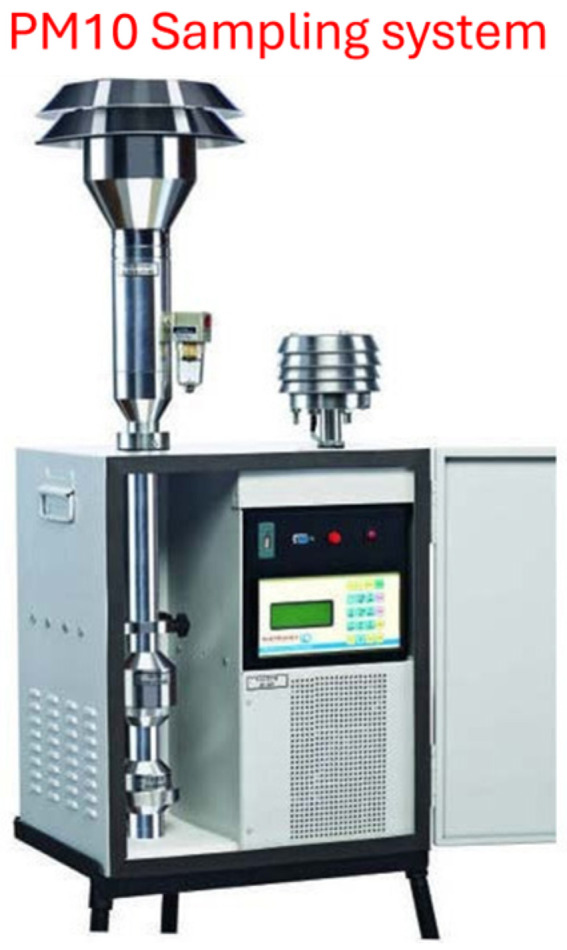
PM10 sampling system.

PM10 samples were collected with the Environnement S.A PM 162 M (Environnement, Poissy Cedex, France) and FAI Instruments Swam 5 and Swam 5 Dual Channel high volume samplers (FAI Instruments, Rome, Italy), on Whatman 1851-047 47 mm quartz microfiber filters (TISCH, Ohio, USA) (Figure 2). Instruments installed at the collection sites sampled for 24 h at a rate of 2.3 m^3^·h^−1^ [29,30,31]. In detail, one filters package (each sample ID), with thirty daily quartz discs, was collected monthly for each sampling point, for a period of one year (2016) and a total of twelve filters packages. The quartz filters, mounted in specific holders, were stored refrigerated, in the dark, before their analysis.

At the laboratory, each filter was punched to obtain a punch of 50 mm^2^ section, used for the inductively coupled plasma mass spectrometry (ICP-MS) heavy metals analysis.

### 2.2. Heavy Metals Analysis

**Figure 3:**
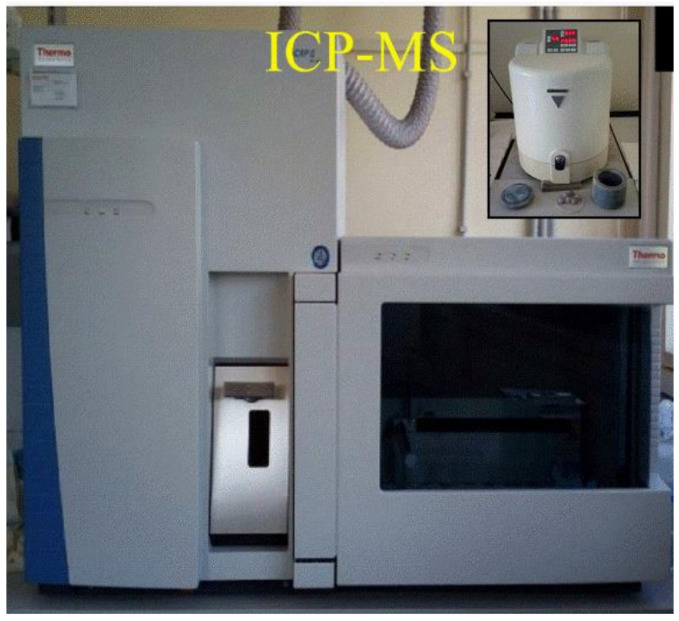
ICP-MS experimental setup.

The concentration of Cd, Ni, and Pb was obtained through ICP-MS analysis using a Thermo Scientific iCAP Qc (Thermo Scientific, Waltham, MA, USA) (Figure 3). 

In detail, a quartz microfiber punch of 50 mm^2^ section (one for each investigated filter), together with 2 mL of ultrapure (67%) HNO_3_ and 1 mL of distilled water were directly introduced into a quartz insert and then subsequently directly introduced into a 100 mL TFM vessel. An additional quantity of liquid, 5 mL of distilled H_2_O and 5 mL of H_2_O_2_ (30%), was placed directly into the 100 mL TFM vessel, around the quartz insert, to a depth equal to the height of the liquid inside the quartz insert. Acid digestion was performed using a Milestone microwave unit system (Milestone, Bergamo, Italy), Ethos touch control, in three steps: 15 min at 1000 W and 200 °C; 10 min at 700 W and 200 °C; 10 min cooling [32]. After cooling, insert contents were filtered and filled up to 50 mL with distilled H_2_O in a 50 mL perfluoroalkoxy-copolymer (PFA) Class A volumetric flask. The sample introduction system consisted of a Peltier cooled (3 °C) baffled cyclonic spray chamber, PFA nebulizer, and quartz torch with a 2.5 mm i.d. removable quartz injector. The instrument was operated in a single collision cell mode, with kinetic energy discrimination (KED), using pure He as the collision gas. All samples were presented for analysis using a Cetac ASX-520 (Thermo Scientific, Waltham, MA, USA). The iCAP Qc ICP-MS was operated in a single KED mode using the following parameters: 1550 W forward power; 0.98 L/min nebulizer gas; 0.8 L min^−1^ auxiliary gas; 14.0 L min^−1^ cool gas flow; 4.5 mL min^−1^ collision cell gas He; 45 s each for sample uptake/wash time; optimized dwell times per analyte (0.01 s); one point per peak and three repeats per sample [33].

**Figure 4:**
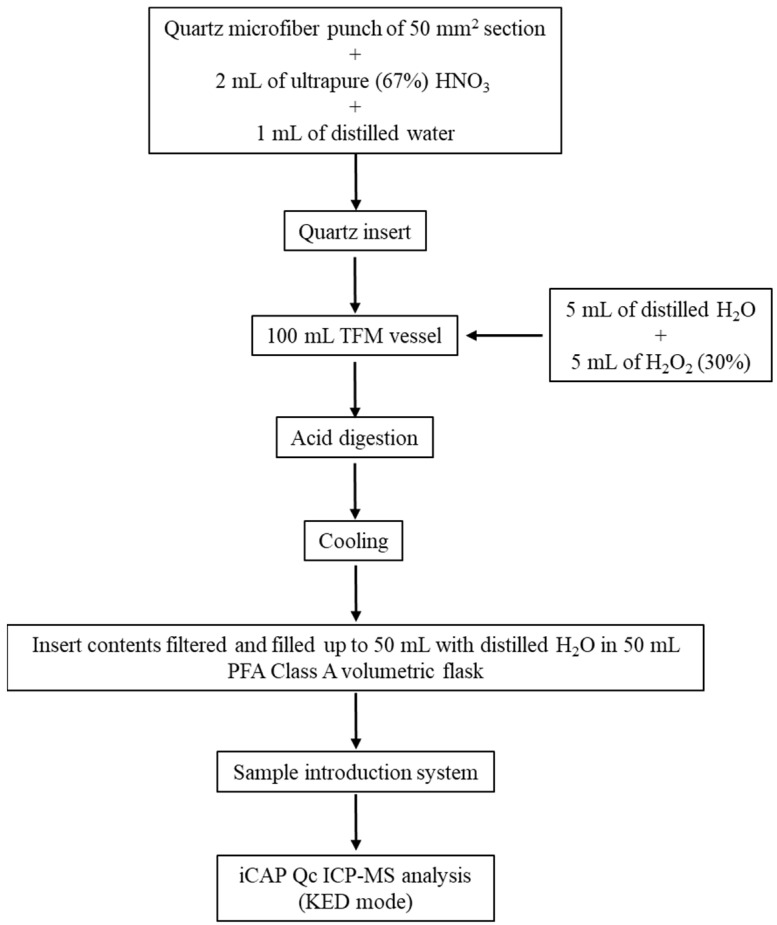
A flowchart showing the steps to perform the sample preparation and ICP-MS analysis.

A flowchart showing the steps to perform the sample preparation and analysis is reported in Figure 4.

### 2.3. Health Risk Assessment

The daily exposure (D) to heavy metals via PM10 was calculated for the three main routes of exposure: direct ingestion (D_ing_), inhalation (D_inh_), and dermal absorption to skin-adhered particles (D_dermal_), according to the US Environmental Protection Agency guidance [34]:

(1) $$D_{ing}=C\times \frac{IngR\times EF\times ED}{BW\times AT}\times CF1$$

(2) $$D_{inh}=C\times \frac{InhR\times EF\times ED}{PEF\times BW\times AT}$$

(3) $$D_{dermal}=C\times \frac{SA\times SL\times ABS\times EF\times ED}{BW\times AT}\times CF1$$

where C (ppm) is the heavy metals concentrations in analyzed samples; IngR (mg·day^−1^) is the conservative estimates of particulate ingestion rates [35]; InhR (m^3^·h^−1^) is the inhalation rate [35]; EF (h year^−1^) is the exposure frequency [34]; ED (years) is the exposure duration [34]; BW (kg) is the body weight [35]; AT (days) is the averaging time [34]; PEF is the particle emission factor (m^3^·kg^−1^) [34]; SA (cm^2^) is the exposed skin area [35]; SL (mg·cm^−2^) is the skin adherence factor [35]; ABS is the dermal absorption factor [34]; and CF1 is the unit conversation factor [34]. Numeric values of the above-mentioned parameters, for adults and children, are reported in Table 2.

The potential non-carcinogenic risk for each heavy metal was estimated using the hazard coefficient (HQ) [36], that, for the three main routes of exposure, was calculated as a ratio of daily exposure (D) to a reference dose of each metal (RfD) [35]:

(4) $${HQ}_{k}=\frac{D_{k}}{RfD}$$

where k is ingestion, inhalation, or dermal route. The total hazard index (HI) of each heavy metal for all routes of exposure was calculated as follows [37]:

(5) $$HI={HQ}_{ing}+{HQ}_{inh}+{HQ}_{dermal}$$

The carcinogenic risk for potential carcinogenic metals was calculated by multiplying the doses by the corresponding cancer slope factor (SF) [38]:

(6) $$Risk=\sum_{k=1}^{n}D_{k}\times {SF}_{k}$$

The carcinogenic ingestion, inhalation, and dermal SFs were provided from the Integrated Risk Information System [39]. Moreover, k is the route of exposure (ingestion, inhalation, or dermal path). The total cancer risk (Risk_total_) of potential carcinogens was calculated as the sum of the individual risk values:

(7) $${Risk}_{total}={Risk}_{ing}+{Risk}_{inh}+{Risk}_{dermal}$$

## 3. Results and Discussion

### 3.1. Heavy Metals Concentration

The air concentration of PM10, deposited on the quartz microfiber filters collected, was directly measured at the sampling sites by using the collecting instruments installed there. The collected data showed seasonal trends with higher concentrations in cold and dry periods than in warm and wet periods. The difference between warm and cold seasons may be caused by the relatively stable energy consumption [40]. Moreover, the average concentration of PM10 in outdoor air for the entire sampling period was about 20 µg·m^−3^ at all sampling sites, lower than the threshold value set by the Italian Legislation (40 µg·m^−3^) [41] and in good agreement with levels typical of most European cities (range: 8.50–29.30 µg·m^−3^ [42]). The concentration of PM10 is markedly associated with natural origin (soil erosion, marine and biogenic aerosols, volcanic emissions, long-distance transport of sand) and/or anthropogenic (heating, industries, traffic, etc.) phenomena [43].

The annual average concentration of the three investigated heavy metals in the analyzed PM10 samples is reported in Table 3.

**Figure 5:**
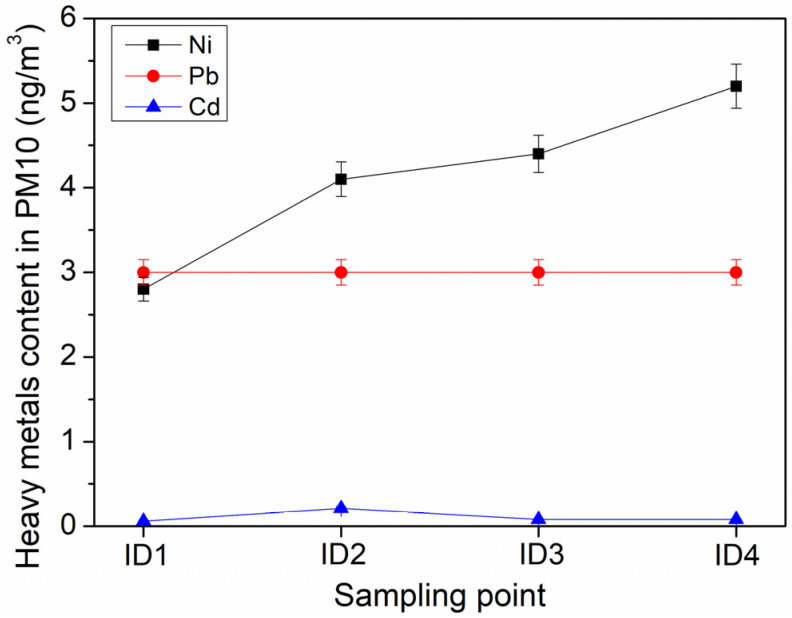
A plot showing the variation of the results.

It is worth noting that the annual average concentrations decrease as follows Ni > Pb > Cd, except for the sampling site ID1, as shown in Figure 5.

Moreover, in all analyzed samples, metal concentrations are lower than the limit values (i.e., 5 ng·m^−3^ for Cd, 20 ng·m^−3^ for Ni, and 0.5 µg·m^−3^ for Pb) reported by the Italian Legislation [41], thus excluding the presence of these heavy metals as pollutants in the analyzed PM10 samples.

### 3.2. Health Risk Assessment

In order to evaluate the impact of heavy metals in PM10 on the health of children and adults, the hazard index and the total cancer risk index were estimated. In particular, Table 4 reports the obtained results for HI, together with reference doses (RfD) and hazard coefficients (HQ_k_), for each sampling point.

Obtained results show that, for children, the total hazard index is 4.1 × 10^−11^, 5 × 10^−11^, 4.5 × 10^−11^, and 4.6 × 10^−11^ for the sampling points ID1, ID2, ID3, and ID4, respectively. All these values are less than the safety limit, HI < 1 [38], thus indicating a negligible non-carcinogenic risk due to the presence of the investigated heavy metals in the analyzed PM10 samples. Notably, the highest value for the total hazard coefficient was obtained for the ingestion pathway (3.9 × 10^−11^, 4.7 × 10^−11^, 4.3 × 10^−11^, and 4.4 × 10^−11^ for the sampling points ID1, ID2, ID3, and ID4, respectively). Therefore, the ingestion pathway represents the highest risk, followed by dermal contact (1.6 × 10^−12^, 3.4 × 10^−12^, 1.8 × 10^−12^, and 1.9 × 10^−12^ as total hazard coefficients for the sampling points ID1, ID2, ID3, and ID4, respectively), while the inhalation pathway represents the lowest risk (total hazard coefficients of 1.1 × 10^−15^, 1.5 × 10^−15^, 1.5 × 10^−15^, and 1.7 × 10^−15^ for the sampling points ID1, ID2, ID3, and ID4, respectively). Finally, Pb represented the highest contribution to the total HI value for children among the investigated heavy metals.

For adults, the total HI was 3.1 × 10^−12^, 4.5 × 10^−12^, 3.4 × 10^−12^, and 3.5 × 10^−12^ for the sampling points ID1, ID2, ID3, and ID4, respectively. These results are similar to those obtained for children, as the dominant exposure pathway was ingestion (total hazard coefficients equal to 2.3 × 10^−12^, 2.7 × 10^−12^, 2.5 × 10^−12^, and 2.6 × 10^−12^ for the sampling points ID1, ID2, ID3, and ID4, respectively). Total HQ values for dermal contact were lower (7.9 × 10^−13^, 1.8 × 10^−12^, 9.4 × 10^−13^, and 9.5 × 10^−13^ for the sampling points ID1, ID2, ID3, and ID4, respectively), and total HQ values were very low for inhalation (3.3 × 10^−16^, 4.4 × 10^−16^, 4.4 × 10^−16^, and 4.9 × 10^−16^ for the sampling points ID1, ID2, ID3, and ID4, respectively).

With reference to the carcinogenic risk to human health through exposure to heavy metals in the analyzed PM10 samples, it was calculated for both children and adults and summarized in Table 5.

Notably, the obtained results for the total cancer risk index are lower than the threshold limit of 1 × 10^−4^ in all cases (i.e., 9.3 × 10^−14^, 1.4 × 10^−13^, 1.4 × 10^−13^, and 1.7 × 10^−13^ for the sampling points ID1, ID2, ID3, and ID4, respectively, for children, and 5.4 × 10^−15^, 7.9 × 10^−15^, 8.4 × 10^−15^, and 1 × 10^−14^ for the sampling points ID1, ID2, ID3, and ID4, respectively, for adults) [38]. Given the above, the carcinogenic risk caused by Cd, Ni, and Pb in the PM10 samples can be considered to be negligible. Finally, similar to HI values, the total cancer risk index for children is higher than that for adults.

## 4. Conclusions

This paper reported the quantitative analysis results of heavy metals content in airborne particulate matter (PM) with a diameter lower than 10 µm (PM10) for four selected sampling sites covering the entire Calabria region, southern Italy. Obtained results show the following: (i) the annual average concentration of the investigated heavy metals in the PM10 samples decreases in the order Ni > Pb > Cd and (ii) concentration values are lower than the limit values reported in the Italian legislation in all cases, thus excluding the presence of Cd, Ni, and Pb as pollutants in the analyzed samples.

Moreover, the health risk was assessed through the calculation of the hazard index and the calculation of the total cancer risk index, for potential non-carcinogenic and carcinogenic risks, respectively, and, notably, both indices were lower than the safety limits, thus indicating negligible health risks.

Finally, this study had some limitations associated with the limited number of sampling points and tested heavy metals. Therefore, the following will be conducted in the near future: (i) an increase in the number of sampling sites in order to have a denser network more representative of the entire region and (ii) the inclusion of a larger number of metals, based on specific identified sources in the area under study, together with an attempt to relate the carcinogenic potential of the most significant concentrations of heavy metals to the population exposed, after determining the extent of pollution from the investigated metals.

## Figures and Tables

**Table 1:** Sampling points, together with their identification code (IDs) and GPS coordinates.

Sampling Point	GPS Coordinates
ID1	39°18′10″ N 16°15′05″ E
ID2	38°54′51″ N 16°35′09″ E
ID3	38°40′29″ N 16°06′08″ E
ID4	38°06′03″ N 15°38′49″ E

**Table 2:** Data for direct ingestion (D_ing_), inhalation (D_inh_), and dermal absorption to skin-adhered particles (D_dermal_) calculation, for adults and children.

	Adults	Children
IngR (mg day^−1^)	50	200
InhR (m^3^ h^−1^)	2.15	1.68
EF (h year^−1^)	1225	
ED (years)	70	6
BW (kg)	80	18.60
AT (days)	25,550	2190
PEF (m^3^ kg^−1^)	6.80 × 10^8^	
SA (cm^2^)	6840	2550
SL (mg cm^−2^)	0.22	0.27
ABS	0.001	
CF1	10^−6^	

**Table 3:** The annual average content of the three investigated heavy metals (Cd, Ni, and Pb) in the analyzed PM10 samples.

Sampling Point	C_Cd_ (ng·m^−3^)	C_Ni_ (ng·m^−3^)	C_Pb_ (µg·m^−3^)
ID1	0.06 ± 0.02	2.8 ± 1.1	0.003 ± 0.001
ID2	0.21 ± 0.11	4.1 ± 1.7	0.003 ± 0.002
ID3	0.08 ± 0.03	4.4 ± 2.3	0.003 ± 0.001
ID4	0.08 ± 0.03	5.2 ± 2.6	0.003 ± 0.001

**Table 4:** The hazard index (HI), together with reference doses (RfD) and hazard coefficients (HQ_k_), for each sampling point.

Sampling Point	Metal	RfD (ppm Per Day)			Children				Adults			
		Ing	Inhal	Dermal	HQ_ing_	HQ_inh_	HQ_der_	HI	HQ_ing_	HQ_inh_	HQ_der_	HI
ID1	Cd	1 × 10^−3^	1 × 10^−3^	1 × 10^−5^	2.2 × 10^−12^	2.7 × 10^−17^	7.6 × 10^−13^	3 × 10^−12^	1.3 × 10^−13^	8.1 × 10^−18^	3.9 × 10^−13^	5.1 × 10^−13^
	Ni	2 × 10^−2^	2 × 10^−3^	5.4 × 10^−3^	5.1 × 10^−12^	6.3 × 10^−16^	6.5 × 10^−14^	5.1 × 10^−12^	2.9 × 10^−13^	1.9 × 10^−16^	3.3 × 10^−14^	3.3 × 10^−13^
	Pb	3.5 × 10^−3^	3 × 10^−3^	5.25 × 10^−4^	3.2 × 10^−11^	4.6 × 10^−16^	7.3 × 10^−13^	3.3 × 10^−11^	1.9 × 10^−12^	1.4 × 10^−16^	3.7 × 10^−13^	2.2 × 10^−12^
	Σ	-	-	-	3.9 × 10^−11^	1.1 × 10^−15^	1.6 × 10^−12^	4.1 × 10^−11^	2.3 × 10^−12^	3.3 × 10^−16^	7.9 × 10^−13^	3.1 × 10^−12^
ID2	Cd	1 × 10^−3^	1 × 10^−3^	1 × 10^−5^	7.6 × 10^−12^	9.4 × 10^−17^	2.6 × 10^−12^	1 × 10^−11^	4.4 × 10^−13^	2.8 × 10^−17^	1.3 × 10^−12^	1.8 × 10^−12^
	Ni	2 × 10^−2^	2 × 10^−3^	5.4 × 10^−3^	7.4 × 10^−12^	9.2 × 10^−16^	9.5 × 10^−14^	7.5 × 10^−12^	4.3 × 10^−13^	2.7 × 10^−16^	4.8 × 10^−14^	4.8 × 10^−13^
	Pb	3.5 × 10^−3^	3 × 10^−3^	5.25 × 10^−4^	3.2 × 10^−11^	4.6 × 10^−16^	7.3 × 10^−13^	3.3 × 10^−11^	1.9 × 10^−12^	1.4 × 10^−16^	3.7 × 10^−13^	2.2 × 10^−12^
	Σ	-	-	-	4.7 × 10^−11^	1.5 × 10^−15^	3.4 × 10^−12^	5 × 10^−11^	2.7 × 10^−12^	4.4 × 10^−16^	1.8 × 10^−12^	4.5 × 10^−12^
ID3	Cd	1 × 10^−3^	1 × 10^−3^	1 × 10^−5^	2.9 × 10^−12^	3.6 × 10^−17^	1 × 10^−12^	3.9 × 10^−12^	1.7 × 10^−13^	1.1 × 10^−17^	5.1 × 10^−13^	6.8 × 10^−13^
	Ni	2 × 10^−2^	2 × 10^−3^	5.4 × 10^−3^	7.9 × 10^−12^	9.8 × 10^−16^	1 × 10^−13^	8 × 10^−12^	4.6 × 10^−13^	2.9 × 10^−16^	5.1 × 10^−14^	5.1 × 10^−13^
	Pb	3.5 × 10^−3^	3 × 10^−3^	5.25 × 10^−4^	3.2 × 10^−11^	4.6 × 10^−16^	7.3 × 10^−13^	3.3 × 10^−11^	1.9 × 10^−12^	1.4 × 10^−16^	3.7 × 10^−13^	2.2 × 10^−12^
	Σ	-	-	-	4.3 × 10^−11^	1.5 × 10^−15^	1.8 × 10^−12^	4.5 × 10^−11^	2.5 × 10^−12^	4.4 × 10^−16^	9.4 × 10^−13^	3.4 × 10^−12^
ID4	Cd	1 × 10^−3^	1 × 10^−3^	1 × 10^−5^	2.9 × 10^−12^	3.6 × 10^−17^	1 × 10^−12^	3.9 × 10^−12^	1.7 × 10^−13^	1.1 × 10^−17^	5.1 × 10^−13^	6.8 × 10^−13^
	Ni	2 × 10^−2^	2 × 10^−3^	5.4 × 10^−3^	9.4 × 10^−12^	1.2 × 10^−15^	1.2 × 10^−13^	9.5 × 10^−12^	5.5 × 10^−13^	3.5 × 10^−16^	6.1 × 10^−14^	6.1 × 10^−13^
	Pb	3.5 × 10^−3^	3 × 10^−3^	5.25 × 10^−4^	3.2 × 10^−11^	4.6 × 10^−16^	7.3 × 10^−13^	3.3 × 10^−11^	1.9 × 10^−12^	1.4 × 10^−16^	3.7 × 10^−13^	2.2 × 10^−12^
	Σ	-	-	-	4.4 × 10^−11^	1.7 × 10^−15^	1.9 × 10^−12^	4.6 × 10^−11^	2.6 × 10^−12^	4.9 × 10^−16^	9.5 × 10^−13^	3.5 × 10^−12^

**Table 5:** The cancer slope and risk factors calculated for children and adults, for each sampling point.

Sampling Point	Metal	SF_ing_	SF_inhal_	Children			Adults		
				Risk_ing_	Risk_inh_	Risk_total_	Risk_ing_	Risk_inh_	Risk_total_
ID1	Cd	-	6.3	-	1.7 × 10^−19^	1.7 × 10^−19^	-	5.1 × 10^−20^	5.1 × 10^−20^
	Ni	0.91	8.4 × 10^−1^	9.2 × 10^−14^	1.1 × 10^−18^	9.2 × 10^−14^	5.4 × 10^−15^	3.1 × 10^−19^	5.4 × 10^−15^
	Pb	8.5 × 10^−3^	4.2 × 10^−2^	9.5 × 10^−16^	5.8 × 10^−20^	9.5 × 10^−16^	5.5 × 10^−17^	1.7 × 10^−20^	5.5 × 10^−17^
	Σ	-	-	9.3 × 10^−14^	1.3 × 10^−18^	9.3 × 10^−14^	5.4 × 10^−15^	3.8 × 10^−19^	5.4 × 10^−15^
ID2	Cd	-	6.3	-	5.9 × 10^−19^	5.9 × 10^−19^	-	1.8 × 10^−19^	1.8 × 10^−19^
	Ni	0.91	8.4 × 10^−1^	1.3 × 10^−13^	1.5 × 10^−18^	1.3 × 10^−13^	7.8 × 10^−15^	4.6 × 10^−19^	7.8 × 10^−15^
	Pb	8.5 × 10^−3^	4.2 × 10^−2^	9.5 × 10^−16^	5.8 × 10^−20^	9.5 × 10^−16^	5.5 × 10^−17^	1.7 × 10^−20^	5.5 × 10^−17^
	Σ	-	-	1.4 × 10^−13^	2.2 × 10^−18^	1.4 × 10^−13^	7.9 × 10^−15^	6.5 × 10^−19^	7.9 × 10^−15^
ID3	Cd	-	6.3	-	2.3 × 10^−19^	2.3 × 10^−19^	-	6.8 × 10^−20^	6.8 × 10^−20^
	Ni	0.91	8.4 × 10^−1^	1.4 × 10^−13^	1.6 × 10^−18^	1.4 × 10^−13^	8.4 × 10^−15^	4.9 × 10^−19^	8.4 × 10^−15^
	Pb	8.5 × 10^−3^	4.2 × 10^−2^	9.5 × 10^−16^	5.8 × 10^−20^	9.5 × 10^−16^	5.5 × 10^−17^	1.7 × 10^−20^	5.5 × 10^−17^
	Σ	-	-	1.4 × 10^−13^	1.9 × 10^−18^	1.4 × 10^−13^	8.4 × 10^−15^	5.7 × 10^−19^	8.4 × 10^−15^
ID4	Cd	-	6.3	-	2.3 × 10^−19^	2.3 × 10^−19^	-	6.8 × 10^−20^	6.8 × 10^−20^
	Ni	0.91	8.4 × 10^−1^	1.7 × 10^−13^	2.0 × 10^−18^	1.7 × 10^−13^	9.9 × 10^−15^	5.8 × 10^−19^	9.9 × 10^−15^
	Pb	8.5 × 10^−3^	4.2 × 10^−2^	9.5 × 10^−16^	5.8 × 10^−20^	9.5 × 10^−16^	5.5 × 10^−17^	1.7 × 10^−20^	5.5 × 10^−17^
	Σ	-	-	1.7 × 10^−13^	2.2 × 10^−18^	1.7 × 10^−13^	1 × 10^−14^	6.7 × 10^−19^	1 × 10^−14^

## Data Availability

Data are contained within the article.
